# Test UHCJ20m—Measurement Procedure Standardization and Metric Characteristics Determination

**DOI:** 10.3390/s20143971

**Published:** 2020-07-17

**Authors:** Aleš Dolenec, Ivan Milinović, Vesna Babić, Dražan Dizdar

**Affiliations:** 1 Faculty of Sport, University of Ljubljana, Gortanova ulica 22, 1000 Ljubljana, Slovenia; 2 Faculty of Economics & Business, University of Zagreb, Trg J.F. Kennedy 6, 10000 Zagreb, Croatia; imilinovic@efzg.hr; 3 Faculty of Kinesiology, University of Zagreb, Horvaćanski zavoj 15, 10000 Zagreb, Croatia; vesna.babic@kif.unizg.hr (V.B.); drazan.dizdar@kif.unizg.hr (D.D.)

**Keywords:** explosive strength, reliability, homogeneity, sensitivity, sprinting, unilateral horizontal cyclic jumps

## Abstract

The purpose of the research study was to standardize the measurement procedure and determine the reliability, homogeneity, and sensitivity of a 20 m unilateral horizontal cyclic jump test (UHCJ20m) whose intentional (assumed) measurement aim is the lower extremities’ explosive strength. The subject sample consisted of 31 students from Zagreb University (20.68 ± 1.96 years of age, height 185.16 ± 7.19 cm, body mass 79.48 ± 9.23 kg) actively involved in various sports events. The UHCJ20m test was performed three times using a dominant (take-off) leg with an active rest of 15 min between the repetitions. The results showed that the UHCJ20m test had satisfactory sensitivity and a very high reliability: Cronbach α = 0.95, intraclass correlation coefficient (ICC) = 0.94 and homogeneity average intertrial correlation (AVR) = 0.88. Future research studies should be aimed at determining the metric characteristics of the UHCJ20m test with a population of athletes in sports characterized by start acceleration and maximum speed running.

## 1. Introduction

In general, motor tests are used to assess latent motor abilities (power, various types of strength, flexibility, speed, coordination, etc.) that are relevant for athletes of all age groups in certain sports and events. Various sets of tests, or measurement of certain candidates’ characteristics, enable talent identification and their selection for a certain sport or sports discipline, monitoring of their athletic development [1] and evaluation of the training process. When the interest of professionals of kinesiology and sport practitioners is focused on sprinting, then the most relevant factors become the area of motor tests and their application in the prediction of sprinting performance, aside from anthropometrical measurements and the determination of relationships between individual athletes’ dimensions (characteristics and abilities) and sprint performance. Even though in this research body height and body mass were measured, they were only used for description of the samples. According to some of the literature, some of these measures can have an influence on the jump results. Excess body mass has a negative effect on the results of the jump [2]. However, with the vertical jump, longer lower limbs [3], and body height [4] have a positive effect. Some authors [5] believe that those variables are not reliable predictors for a successful test of power or for testing explosive leg power.

Motor tests, used to evaluate the current preparedness level of athletes, can be undertaken either in a laboratory or in the field [6]. For preparedness testing purposes, tests are selected according to the following criteria: specificity, standardization, reliability, and validity [7]. Each type of testing, either in a laboratory or field, has its own advantages and disadvantages. Laboratory motor tests, or test batteries, are used frequently due to their independence of weather conditions and high reliability, but they are also often demanding an in organizational, financial and technical sense, while field tests are often less reliable and precise but their application in practice is simple and cheaper [8]. When applying tests, different measuring devices can be used that affect the measured values of individual variables. Depending on the test, timing can be done manually with a stopwatch, electronically with a measuring system such as single or dual-beam photocells [9], OPTOJUMP [10], a photo finish [11], Chip timing [12,13], a GPS system [14,15] and other electronic systems. Photocells are most commonly used in research to measure running time, and manual measurement with a stopwatch still predominates in athlete training. The repeatability and reliability of measuring the running time manually with a stopwatch or photocells is the same, except that with manual measurement with a stopwatch there is a certain systematic error compared to photocells [16,17,18].

Jumps/hops are complex movement patterns that require complex motor coordination of the whole body, particularly the upper and lower extremities [19], so they can be used as tests for the prediction of certain sprint running phases, such as start acceleration, maximal speed running, and agility [20,21,22,23,24,25,26], as well as in almost all individual and team sports in which the capability of speed-strength impulse production is required from athletes [27]. It is of great importance in training/coaching practice to use specific motor tests intended to diagnose the exact traits of the athletes, subject to the condition that they have met the criteria of reliability and validity. Explosive strength (power) of a jumping type, that is the athletes’ jumping ability, has been the subject of assessment in many available research studies investigating different types of jumps (bilateral, unilateral, horizontal, and vertical). The review of the available literature has revealed reliability coefficients for the vertical jumps ranging over the interval of an intraclass correlation coefficient (ICC) = 0.71–0.98 [20,21,28,29,30], whereas for the horizontal jumps these values are ICC = 0.87–0.99 [19,20,21,28,30]. However, we have found that the available research studies have not investigated the issue of unilateral horizontal jumps to any great extent. The most common unilateral horizontal jumps investigated were the unilateral horizontal countermovement jump—*HCMJ* [20,29,31,32], the 6 m timed hop test [33,34,35,36], the unilateral standing horizontal jump—*SLJ* [26,37,38], the unilateral standing drop horizontal jump—*SLDJ* [39], the standing triple horizontal jump—*TLJ* [26,29,37,38,40], standing five jumps—*SFJ* [41], standing ten jumps—*STJ* [41], and unilateral 30 m horizontal cyclic jumps—*UHCJ30m* [42].

The UHCJ20m test is frequently used in athletes’ practice, although the available literature offers no guidelines on the standardization of the measurement procedure or record of the determination of its metric characteristics. By reviewing the available published research studies, the only thing that was found was some mentions of similar motor tests, in which the performance was expressed in time units. These motor tests were the 6 m timed hop test [33,34,35,36] and the UHCJ30m [42]. The 6 m timed hop test is used in the field of medicine in the evaluation of patients’ recovery after anterior cruciate ligament surgery, and no papers have been found in the literature confirming the use of the test in sports. The difference between gifted and ungifted girls for sprinting can be determined by the results achieved in the UHCJ30m test [42]. Although the UHCJ20m test is classified as a test for the assessment of the explosive strength (power) of legs, the goal of the test is to attain the maximal possible speed, that is, to cover a 20 m distance in the shortest possible time. Due to that time component, it is feasible to assume that the goal of this test is not the generation of maximal muscle force in the time unit but athletes being tested should successfully balance take-off strength, take-off direction, speed, frequency of jumps, and coordination maintenance in order to achieve the most efficient and fastest ambulatory progression. Therefore, the aim of this research study was to standardize the measurement procedure and to determine the reliability, homogeneity, and sensitivity of the UHCJ20m test.

## 2. Materials and Methods

### 2.1. Subject Sample

The convenience subject sample consisted of 31 young men who represented the student population of the University of Zagreb, who were of a good health status and involved in different sports and recreational activities, however none were members of the specific sprinter population. On average, subjects were 20.68 ± 1.96 years of age with a body height of 185.16 ± 7.19 cm and a body mass of 79.48 ± 9.23 kg. They were familiarized with the purpose of the experiment and expectations of their participation, after which they signed the consent form with all the key facts relevant to the experiment.

### 2.2. Test

Since we had found no data in any scientific publications on the use of this motor test for the assessment of the explosive strength of legs, our first task was to standardize the test. The description of the test is presented in Table 1. The possibilities of conducting this test in either a laboratory or in the field are listed. A simple way of conducting this test is to provide a 30 m long straight, smooth, and hard surface on which a 20 m distance is marked, a stopwatch, and any type of tool to signal the start. There is also a more complex version of this test (used in this study) which uses the apparatuses of the optical system OPTOJUMP (Microgate, Bolzano, Italy), a BROWER Timing System pair of photocells (Brower Timing System, Draper, USA), and a computer provided with the appropriate software. The use of a manual system (with experienced or inexperienced timers) or an electronic system for measuring results can cause a difference between the results, but the reliability of the obtained results is the same between the systems [17]. It is important, when comparing the progress of athletes, that the same method of measuring results is always used on the same athlete. More complex equipment provides insight into certain kinematic parameters during the task execution while only a stopwatch does not.

The test commences with the subject standing behind the start line in a standing start position, with the jumping leg placed forward. Upon the measurer’s start signal, the subject sets off with the forward leg and with an alternate arm swing (not simultaneous) and continues to perform unilateral jumps thus covering the distance in the shortest possible time. The task is regarded as performed correctly if the subject has met the following basic criteria: he/she has initiated the process of jumping only after the start signal and completed the task by jumping on the same one leg while using the alternate arm swings along the track to the finish line. The task is performed three times and the best result is used as the test outcome. While warming up before the actual test the students have a trial jump test. Between the repetitions, during active rest intervals which are sufficient for a full recovery, the participants perform stretching exercises and light jogging. Subjects who did not follow the test protocol repeated the task after a break of 15 min, if they made a mistake during the performance of the jumps, and in case of an error at the start, the subjects immediately repeated the task.

### 2.3. Experiment Protocol

The task was explained to the subjects in the gymnasium and they were given a consent form to sign, agreeing to their participation in the experiment. For the purpose of the sample’s description, each subject was measured for his body height and mass. Consequently, the subjects took part in a 15–20 min collective warm-up preparation for the task execution, which consisted of running at variable speeds interspersed with various tasks, predominantly a series of horizontal jumps and hops, followed by some stretching exercises. Following the preparation, the task was twice demonstrated to the subjects. For the purposes of the standardization of measurement procedure and the determination of the metric characteristics of the test, the participants executed the task with their dominant (take-off) leg. Horizontal jumps with the dominant leg are usually longer, and the standard deviation is smaller than with jumps with the non-dominant leg [43]. The UHCJ20m test is not intended for use for rehabilitation purposes, so due to stable metric characteristics and rationalization, only the dominant leg was measured.

The test began with the subject positioning himself behind the start line marking the beginning of the 20 m distance. On the command “On your marks”, the subject assumed a standing start position with the dominant (take-off) leg forward, nearer to the start line. On the start signal, marking the initiation of the task execution, the subject performed unilateral horizontal cyclic jumps until the end of the 20 m distance where the photocells recorded the result. The test was executed three times with 15 min active rests between the attempts, during which the subjects performed stretching exercises and jogging.

### 2.4. Data Processing Methods

The basic descriptive parameters were calculated for all the raw data, while the normality of distribution was tested by the Kolmogorov–Smirnov test. To determine the internal metric characteristics of the UHCJ20m test all the three attempts were considered. Reliability was calculated by the method of internal consistency (Cronbach α—reliability coefficient, AVR—average intertrial correlation, ICC—intraclass correlation coefficients, SEM—Standard Error of Measurement, CV—coefficient of variation). Homogeneity was calculated according to an AVR—average correlation among the items, Pearson’s correlation coefficient between the three test measurements, whereas sensitivity was calculated by dispersion measures (results range, standard deviation, and variability coefficient) and by measures of the curvature (skewness and kurtosis). Results were processed using the statistical package Statistica 13.0 at the significance level of *p* ≤ 0.05.

All subjects gave their informed consent for inclusion in the experiment. The study was conducted in accordance with the Declaration of Helsinki, and the protocol was approved by the Ethics Committee of the Faculty of Kinesiology, University of Zagreb (No. 42/2018).

## 3. Results

Descriptive indicators of the variables (Table 2) obtained with the convenience sample of 31 male students were calculated for the purpose of analysis and for the determination of the internal metric characteristics of a new motor test, UHCJ20m. The following parameters are shown: arithmetic mean (M), standard deviation (SD), minimal and maximal result (Min and Max), skewness (Skew), kurtosis (Kurt), variance (V), Kolmogorov–Smirnov test of distribution normality (max D).

Arithmetic means of the test results indicated that the participants on average scored worst in the first test attempt (5.22 ± 0.44 s), whereas the best average result was achieved in the second attempt (5.08 ± 0.40 s). The result of the third measurement was almost at the level of the second measurement result (5.10 ± 0.44 s), whereas the arithmetic mean of the results of all the three measurements was 5.14 ± 0.41 s. The results of all test items, according to the K–S criterion at the p level of *p* ≤ 0.05, did not substantially deviate from normal distribution.

Table 3 shows the coefficients of correlation matrix. It can be concluded that a significant strong positive correlation was obtained between the results of the first and second measurement (r = 0.874), as well as between the first and the third measurement (r = 0.848) in the UHCJ20m test, whereas the highest significant correlation was between the second and third measurement (r = 0.906).

The reliability of three measurements of the UHCJ20m test was determined by the condensation of the results in three ways: by a simple linear combination of the original results (Cronbach α), intraclass correlation (ICC), Standard Error of Measurement (SEM) and coefficient of variation (CV). The reliability results (Table 4) indicate that the composite measuring instrument has a high reliability (Cronbach α = 0.95, ICC = 0.94, SEM = 0.09 and CV = 1.8). The subject who achieved the above average result in one measurement also achieved the above average results in other two measurements. The average correlation between items was AVR = 0.88, which was indicative of a high homogeneity of the measuring instrument. The lower part of Table 4 shows reliability coefficients by items and values of their arithmetic means, variance, standard deviations, correlation of the mentioned item with a simple linear combination of all other items, and the coefficient of the test reliability after omitting certain test items. The second and third measurements showed a higher correlation, so the reliability coefficient had the highest value when the first measurement item had been omitted (Alpha if deleted = 0.95).

## 4. Discussion

Given the fact that test UHCJ20m is used in practice, although the available research studies have shown no records of its metric characteristics or standardized descriptions, it was necessary to define the final form of the test thus standardizing this measuring instrument. The procedure implies defining the object of measurement, the choice of an appropriate test, the choice of stimuli, standardization, and determination of the metric characteristics. The test is used to assess the latent dimension of explosive strength (power) of the lower extremities. The standardization of the measuring procedure implies a precise description of all procedures utilized and conditions under which measurement with a particular measuring instrument is conducted, as well as the results’ scoring method and their validation [44]. All the subjects performed the test three times using their better, dominant (take-off) leg. After the test results had been collected, the metric characteristics of test UHCJ20m were determined.

Three repeated measurements of test UHCJ20m were tested by the Kolmogorov–Smirnov test at the significance level of *p* ≤ 0.05, which confirmed that the results in all the test items did not significantly deviate from normal distribution. The descriptive statistics revealed, on average, the best performances in the second measurement and similar performances in the last measurement, which may indicate that the subjects were still learning the motor task during the first measurement although an average difference in the achieved results was not great. The results of the Pearson correlation coefficients between the measurement items indicate a high correlation between them. It was higher between the second and third measurements compared to the first, which, given that the subjects had a warm-up test, is to assume that they react differently to the stimulus under artificial conditions, so the test should be realized predominantly under real conditions. Given the test is a composite measuring instrument, reliability was determined via the method of internal consistency through the condensation of the results with a simple linear combination of the original results—Cronbach α = 0.95, ICC = 0.94. The high reliability coefficients obtained suggest a reliable measuring instrument. This motor test has reliability values at the level of similar tests used to assess explosive strength of the legs, for instance, the standing triple jump, the standing long jump, the Sargent test, the squat jump, which showed reliability coefficients (Cronbach’s α) in the range of 0.93 to 0.98 [19]. In the publications whose goal was to determine the metric characteristics of motor tests used to assess the explosive strength (power) of legs using unilateral and bilateral jumps in either vertical or horizontal direction, the test–retest method was employed to determine reliability. The test–retest method implies the application of the same motor test to testing the same group of subjects at two different time points; the correlation between the results of the first and repeated measurement was considered the reliability coefficient [44]. The reliability obtained by this method for horizontal jumps, according to the results of some research studies, was in the range of ICC = 0.89–0.99 [20,28,29,32,41]. The test presented here has a very high homogeneity although the first item (first measurement) had a somewhat weaker correlation compared to the second and third item, therefore it is suggested at least one practice trial is used. An average correlation between the test items meets the homogeneity criterion and is AVR = 0.88, indicating that the results achieved by the subjects were in all the items dependent on the same measurement object. Measures of dispersion and the shape of data distribution in the repeated measurements displayed an adequate sensitivity, thus allowing for the differentiation among the subjects according to the measurement object. Therefore, it is feasible to conclude that motor test UHCJ20m has satisfactory internal metric characteristics (reliability, homogeneity, and sensitivity) for the population of student athletes of the University of Zagreb. Having in mind more efficient and more practical testing athletes, our recommendation for the next validation of this test, as well as for its practical use, is to decrease the rest time interval (from 15 min to 5 min) between the attempts since five minutes seems a sufficient time during which subjects can regenerate for the next test task performance. A limitation of this research could be the complexity of unilateral speed jumps, whose performance requires good technique and good foundations that team sports athletes do not possess to such an extent. The test would be more suitable for the sprint population, which represents a more practical application for them, but also great training opportunities [45].

## 5. Conclusions

The results of this research study demonstrate that the motor test UHCJ20m has very high coefficients of reliability, independent of the method used to determine the final results in the test (assessment of real result) and, consequently, using more complex methods of real result evaluation did not yield significantly higher reliability. Therefore, it is suggested to use the simplest way of condensation (real result evaluation)—by arithmetic means of the original results. Further, with regard to a very high reliability coefficient, and in order to achieve even greater test efficiency, it is possible to perform the test task twice and use the better result as the final result. By doing so, more time would be saved while conducting the test with the reliability remaining. The reliability calculation of the composite measuring instrument from the three measurement items is on the level of the results for other tests used to assess explosive leg strength that can be found in scientific literature. The reliability of horizontal jumps in the population of athletes of different sports ranges from 0.89 to 0.97 [20,21,29]. Additionally, the test has a remarkably high homogeneity considering that the first item (first measurement) has a weaker correlation compared to the second and third measurement; it is suggested therefore that at least one practice trial be used. Lastly, this test has satisfactory sensitivity for the population of student athletes from Zagreb University. In any case, the UHCJ20m is a specific test since the available literature hardly mentions the tests of unilateral horizontal cyclic jumping ability measured in time units (except for the 6 m timed hop test and UHCJ30m test). To perform well in the test, athletes should be apt and dexterous to achieve the greatest speed possible with regard to the necessity of balancing between the length and frequency of the jumps, the latter two being under the direct influence of other parameters such as ground contact time and flight time. In the performance of the test, the subjects certainly compensated in different proportions for a lack of explosive power, speed or coordination of movements, which are the basic abilities required by the performance, but the authors at this stage of the research did not investigate the quantitative indicators of these abilities; nevertheless, this should be rectified in the future. Further research studies should focus on determining the metric characteristics of the test with a population of athletes in sports dominated by start acceleration and maximal speed running and justifying its practical application to talent identification and selection, monitoring of athletes’ development, as well as to monitoring and evaluation of the training process. This is especially pronounced if the testing is conducted with a small number of subjects where the rest interval between repetitions of the task would be a minimum of 5 min, and in the case of testing a larger number of subjects, this interval should not exceed 15 min including some activity. Since the test is used in sprint training processes, the authors have every intention to determine the prognostic validity of the test on the success of sprint running and provide justification for the use of UHCJ20m in practice.

## Figures and Tables

**Table 1:** Representation of the standardization of a motor 20 m unilateral horizontal cyclic jump test (UHCJ20m).

Test: 20-m Unilateral Horizontal Cyclic Jumps	
TEST CODE	UHCJ20m
MEASUREMENT PURPOSE	Explosive Strength of Legs (Elastic Strength)
NECESSARY EQUIPMENT	Laboratory: A straight, smooth, and hard surface with a minimum of 30 m in length and 1-m in width; the start and finish lines are marked 20 m apart; BROWER Timing System photocells; OPTOJUMP optical system; and computer with an appropriate software; a starting signal apparatus. Field: A straight, smooth, and hard surface with a minimum of 30 m in length and 1-m in width; the start and finish lines are marked 20 m apart; a stopwatch; a starting signal apparatus.
TEST EXECUTION DESCRIPTION	The subject is stands behind the start line in the position for a standing start with one leg nearer to the start line. After the measurer’s command “On your marks” and the start signal, the subject begins and the task execution and sets off with the front leg and by the alternate arm swing (not simultaneous). The subject covers the distance in the shortest possible time using the same leg jumps. Correctness criteria: the task execution begins only after the start signal and the task is completed via the marked distance to the finish line by jumping on the same one leg using the alternate arm swings. The task is executed three times. Active rest intervals (stretching and light jogging) between the attempts should ensure full recovery of the participant.
INSTRUCTIONS TO SUBJECTS	The task is demonstrated and elaborated: “This is a test used to assess explosive strength of the legs. Your goal is to cover the 20 m distance using unilateral jumps in the shortest possible time starting from the standing start position and setting off with the front leg in the start position after the measurer’s command “On your marks” and the start signal. You can use alternate arm swings (as in running). Try to find your best balance between your jump length and the frequency in your efforts to attain the greatest speed of movement possible.
RESULTS DETERMINATION	The result is time needed to cover the 20 m distance using unilateral horizontal cyclic jumps. The task is performed three times. The best attempt is recorded as the test outcome. (If any a sophisticated additional equipment is employed in a laboratory, the kinematic measures such as the jump length and the jump frequency, speed of movement, number of jumps, etc. are used along with the end results). The result is expressed in tenths of a second.

**Table 2:** Descriptive indicators of the UHCJ20m test items.

N = 31/Variable	M	Min	Max	V	SD	Skew	Kurt	Max D
UHCJ20m1 (s)	5.22	4.39	6.63	0.19	0.44	1.13	2.62	0.07
UHCJ20m2 (s)	5.08	4.38	6.35	0.16	0.40	0.84	2.42	0.05
UHCJ20m3 (s)	5.10	4.19	6.52	0.20	0.44	0.83	2.74	0.04
UHCJ20m1-3 (s)	5.14	4.32	6.50	0.17	0.41	1.09	3.35	0.10

K-S0.05 = 0.24. Legend: N—number of subjects, M—arithmetic mean, Min—minimal result, Max—maximal result, V—variance, SD—standard deviation, Skew—skewness, Kurt—kurtosis, UHCJ20m1, 2, 3—measurements of 20m unilateral horizontal cyclic jumps, UHCJ20m1–3—arithmetic mean of three test measurements, max D—maximal deviation of the relative cumulative empiric frequency from the relative theoretical frequency.

**Table 3:** Correlation matrix of the repeated measurements in motor test, UHCJ20m.

Variable	UHCJ20m1	UHCJ20m2	UHCJ20m3
UHCJ20m1	1.000		
UHCJ20m2	0.874 *	1.000	
UHCJ20m3	0.848 *	0.906 *	1.000

Legend: UHCJ20m1, 2, 3—measurements of 20 m unilateral horizontal cyclic jumps, * *p* ≤ 0.05.

**Table 4:** Reliability coefficients of the composite measuring instrument 20 m unilateral horizontal cyclic jumps.

	**M±SD**	**AVR**	**Cronbach α**	**ICC**	**SEM**	**CV (%)**
UHCJ20m(s)	5.14 ± 0.41	0.88	0.95	0.94	0.09	1.8
	**M±SD**		**Itm-Tot. Correlation**	**Alpha If Deleted**		
UHCJ20m1(s)	5.22 ± 0.44		0.88	0.95		
UHCJ20m2(s)	5.08 ± 0.40		0.93	0.92		
UHCJ20m3(s)	5.10 ± 0.44		0.90	0.93		

Legend: M—arithmetic mean; SD—standard deviation; AVR—average intertrial correlation; Cronbach α—Cronbach’s alpha reliability coefficients; ICC—intraclass correlation coefficients; SEM—Standard Error of Measurement; CV—coefficient of variation, UHCJ20m1, 2, 3—measurements of 20m unilateral horizontal cyclic jumps, Itm-Total Correlation—item correlation by a simple linear combination of all other items; Alpha if deleted—test reliability coefficient if the mentioned item is omitted.
